# Atomistic Insights into the Influence of High Concentration H_2_O_2_/H_2_O on Al Nanoparticles Combustion: ReaxFF Molecules Dynamics Simulation

**DOI:** 10.3390/molecules29071567

**Published:** 2024-03-31

**Authors:** Xindong Yu, Pengtu Zhang, Heng Zhang, Shiling Yuan

**Affiliations:** 1 Key Lab of Colloid and Interface Chemistry, Shandong University, Jinan 250100, China; 202212213@mail.sdu.edu.cn; 2 School of Chemical Engineering, Shandong Institute of Petroleum and Chemical Technology, Dongying 257061, China; ptzhang@sdipct.edu.cn; 3 Shandong Chambroad Holding Co., Ltd., Binzhou 256500, China

**Keywords:** Al nanoparticles, ReaxFF, combustion, hydrogen peroxide

## Abstract

The combination of Al nanoparticles (ANPs) as fuel and H_2_O_2_ as oxidizer is a potential green space propellant. In this research, reactive force field molecular dynamics (ReaxFF-MD) simulations were used to study the influence of water addition on the combustion of Al/H_2_O_2_. The MD results showed that as the percentage of H_2_O increased from 0 to 30%, the number of Al-O bonds on the ANPs decreased, the number of Al-H bonds increased, and the adiabatic flame temperature of the system decreased from 4612 K to 4380 K. Since the Al-O bond is more stable, as the simulation proceeds, the number of Al-O bonds will be significantly higher than that of Al-H and Al-OH bonds, and the Al oxides (Al[O]_x_) will be transformed from low to high coordination. Subsequently, the combustion mechanism of the Al/H_2_O_2_/H_2_O system was elaborated from an atomic perspective. Both H_2_O_2_ and H_2_O were adsorbed and chemically activated on the surface of ANPs, resulting in molecular decomposition into free radicals, which were then captured by ANPs. H_2_ molecules could be released from the ANPs, while O_2_ could not be released through this pathway. Finally, it was found that the coverage of the oxide layer reduced the rate of H_2_O_2_ consumption and H_2_ production significantly, simultaneously preventing the deformation of the Al clusters’ morphology.

## 1. Introduction

In recent years, with the development of missiles and aerospace technologies, it has become essential to develop a green and high-efficiency propulsion system [1,2,3,4]. Conventional propellant fuels are primarily consisting of hydrazine (N_2_H_4_) and methylated derivatives of hydrazine, etc. However, their disadvantages, such as extremely high toxicity, carcinogenicity, high volatility, and risk of explosion, increase the cost and environmental impact of these propellants during production, storage, and transportation [5,6]. Therefore, the development of a more eco-friendly, lower-cost, and higher-performance propellant composition has been a topical issue in space science.

H_2_O_2_ has been used as a monopropellant and a non-volatile oxidizer for rockets since the 1940s [7,8,9]. Its complete decomposition releases only water vapor, oxygen, and heat, with minimal environmental impact. This propellant offers the aerospace industry an attractive approach to using green propellants with greatly reduced toxicity and low storage and disposal costs [10,11]. In monopropellant rocket systems, the decomposition and combustion efficiency of H_2_O_2_ can be improved by using multiphase catalysts [12]. In bipropellant rocket systems, high concentrations of hydrogen peroxide are used as oxidizers for different fuels (hydrocarbons, kerosene, alcohols, etc.) to increase specific impulse and develop high-energy, environmentally friendly propellant formulations [13,14,15,16,17,18]. For example, Okninski reported a 3.5% improvement in specific impulse and a 70% gain in density-specific impulse by using 98% H_2_O_2_, compared to motors using N_2_O as an oxidizer [19].

On the other hand, as the third most abundant element in the earth’s crust, aluminum has a very high energy density (30.5 kJ/g), low application costs, and environmentally friendly and non-polluting use. For this reason, it is widely applied in various technological fields, including aerospace technology, automobiles, airplanes, and high-energy materials [20,21,22]. Among the composite propellants, Al powder is often used as a metal fuel to enhance the energy characteristics of propellants. The combination of ANPs with conventional oxidizers, such as ammonium perchlorate (AP), 1,3,5-trinitro-1,3,5-triazinane (RDX), 1,3,5,7-tetranitro-1,3,5,7-tetrazocane(HMX), 2,4,6-trinitrotoluene (TNT), etc. is also very widely used in application research [23,24,25,26,27].

By contrast, solid–liquid hybrid propellants with Al as the fuel and H_2_O_2_ as the oxidizer have been studied relatively little. Above all, solid–liquid hybrid systems are safer for transportation, storage, and operation than solid systems and liquid systems. Meanwhile, their specific impulse is generally higher than that of solid rockets, and the density-specific impulse is higher than that of liquid rockets. Moreover, ANPs can obtain a high exotherm (25.8 kJ/g) as well as yield only clean oxidation products (H_2_O, H_2_, O_2_, and Al_2_O_3_) in the reaction with H_2_O_2_, which is why Al/H_2_O_2_ is a promising bipropellant for development [28,29,30].

Zaseck et al. showed that the size of the aluminum particles was the most dominant factor driving the combustion rate of the bipropellant, with the combustion rate exponent increasing from ∼0.5 to ∼1.0 as the diameter of the Al particles decreased from 12 μm to 3 μm. Furthermore, the concentration of H_2_O_2_ had an important effect on the combustion rate and combustion temperature, compared to the mixing ratio (O/F), which had the least effect on the combustion rate [29]. The effect of H_2_O_2_ on the combustion properties of aluminum-water mixtures was investigated by Sabourin et al. The linear combustion rate increased from 9.6 cm/s to 58 cm/s at 3.65 MPa as the mass fraction of H_2_O_2_ was increased from 0 to 32%, and the flame temperature increased by 600 K as the mass fraction of H_2_O_2_ was increased from 0 to 35% under chemically proportioned conditions [30]. According to research, H_2_O_2_ usually undergoes spontaneous decomposition, and the main product of decomposition is water. Hence, its concentration decreases over time, which has a great impact on the combustion efficiency of the propellant [12]. In addition, ANPs have a higher surface area relative to micron-sized Al particles, which contributes to faster oxidation, while ANPs rapidly form an oxide shell on the surface in the presence of an oxidizing agent, and the core-shell structure (Al@Al_2_O_3_) ultimately influences the adiabatic flame temperature and reaction mechanism of the Al/H_2_O_2_ reaction [31,32]. As a result, understanding the basic combustion mechanism of ANPs with H_2_O_2_ and the influence of the percentage of water content on the reaction is crucial for enhancing propulsion performance and energy efficiency.

Experimentally revealing the aforementioned combustion pathways, particularly at the molecular/atomic level, is still quite difficult. Yet ReaxFF-MD simulations provide much richer molecular/atomic details and have been widely used as an alternative to combustion and pyrolysis experiments, etc. [33,34,35,36,37,38]. For example, ReaxFF-MD simulations have successfully captured the atomic-level mass transfer and the reactive processes of the oxidation of ANPs under high-temperature and high-pressure oxygen atmospheres, which reveal the detailed mechanism of the oxidation of ANPs [39]. Zhao et al. revealed the reaction mechanism of molten Al nano-droplets (ANDP) with H_2_O vapor at high temperatures from an atomic perspective using ReaxFF. The influence of temperature, ANDP particle size, and water vapor concentration on ANDP combustion was also considered [40]. Recently, the influence of ANPs as additives on the thermal decay mechanism of energetic materials (EMs) and the evolution of ANPs during the thermal decomposition of EMs have also been explored through reactive molecular dynamics simulations [41,42].

Here, this work will use ReaxFF-MD simulations to investigate the influence of adding different proportions of H_2_O on the combustion of Al/H_2_O_2_ by analyzing the number of bonds, reaction products, intermediates, morphology of ANPs, and reaction trajectories of the whole reaction process. In addition, it aims to explore the combustion mechanism of Al/H_2_O_2_/H_2_O from the atomic perspective. The findings of this study will help to provide a theoretical basis for an in-depth study of Al/H_2_O_2_ combustion and guide the design of subsequent development of binary propulsion systems.

## 2. Results

### 2.1. Reactive Force Field (ReaxFF) Molecular Dynamics

ReaxFF is a molecular dynamics simulation method for chemical reactions, first proposed in 2001 by van Duin et al. [43]. In the model of the reactive force field, the concept of atom type in the classical force field no longer exists, and there is no connectivity between the atoms in the system; instead, at the current moment, connectivity is determined by calculating the bond order (BO) between any two atoms. As chemical bonds are broken and created, the list of atomic connectivity is updated. Thus, it is a molecular force field based on bond order [44,45]. The strength of the ReaxFF force field lies in its ability to handle chemical reaction processes in larger systems on longer time scales and with an accuracy approaching that of quantum chemical calculations. As a result, ReaxFF has been widely used in the field of materials science for more than two decades, in areas such as development and design.

### 2.2. Model Construction

**Figure 1:**
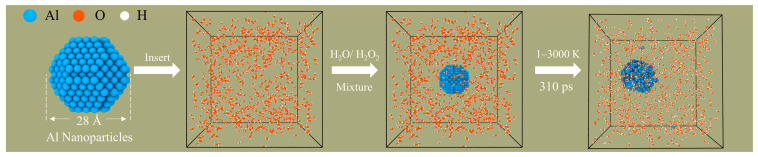
Model building and simulation process (aluminum, oxygen, and hydrogen are blue, orange, and white, respectively).

All of the ReaxFF-MD simulations in this paper were performed in the large-scale atomic/molecular massively parallel simulator (LAMMPS) package [46,47]. This work uses the Al/C/H/O force field parameter set developed by Hong et al. [48]. The force field has been successfully used to reveal the influence of carbon coatings on the oxidation of ANPs and the combustion reaction of ANPs with oxidizing agents (O_2_, H_2_O, H_2_O_2_, etc.) [49,50]. First, an Al particle with a diameter of 2.8 nm and 675 atoms was constructed. The ANPs were then placed in periodic boxes of 10.0 nm × 10.0 nm × 10.0 nm by Packmol, which were surrounded by a random distribution of a predetermined amount of H_2_O_2_/H_2_O molecules in different ratios [51]. The thickness of the vacuum layer between ANPs and H_2_O_2_/H_2_O was set to 1.1 nm to prevent the initial chemisorption of molecules on the Al surface. The model construction process and specific parameters are shown in Table 1 and Figure 1, respectively.

### 2.3. Computational Details and Post-Processing

For the sake of optimizing the system structure, the system was first simulated at 1 K for 10 ps, heated up to 3000 K at a rate of 30 K/ps, and then simulated at 3000 K for 200 ps. The simulations were performed under the canonical ensemble (NVT, where N, V, and T represent the total number of particles, the system volume, and the temperature, respectively), and the temperature of the system was controlled using a Nosé-Hoover thermostat, with a combined time for all simulations of 310 ps. In addition, a microcanonical ensemble (NVE) was performed for each system to simulate the change in temperature of the system in an adiabatic state with 250 ps. The time step for all simulated processes was set to 0.2 fs. Bond breakage and formation were determined by the method of the bond order cutoffs, where the cutoff value was set to 0.3.

According to the previous literature, the set values of the above parameters are reliable [44,48]. High temperature only affects the reaction rate and has little influence on the reaction mechanism in ReaxFF-MD simulations [52]. Hence, we used high-temperature simulations during the combustion process. Since the experimental time is much longer than the simulation time, the number of interatomic collisions is increased by increasing the temperature, which improves the combustion efficiency and reduces the simulation cost. Meanwhile, the evolution of chemical species and the number of bonds in the simulations were post-processed by using a Python program, and the simulation results were visualized with OVITO [53].

## 3. Discussion

### 3.1. Influence of the Addition of H_2_O on the Combustion of ANPs in H_2_O_2_

Propulsion grade H_2_O_2_ has a long history of safe production and application in power and propulsion units, and optimum performance is obtained by using the highest concentration (i.e., 100% H_2_O_2_). Nevertheless, the experimental H_2_O_2_ contains a little water in a reduced concentration due to the compromise between the production cost and the performance obtained and the spontaneous decomposition of H_2_O_2_ over time. Therefore, we established four systems of Al/H_2_O_2_/H_2_O with different percentages, containing 0, 10%, 20%, and 30% of water, respectively, and carried out combustion simulations at 310 ps for the four systems.

**Figure 2:**
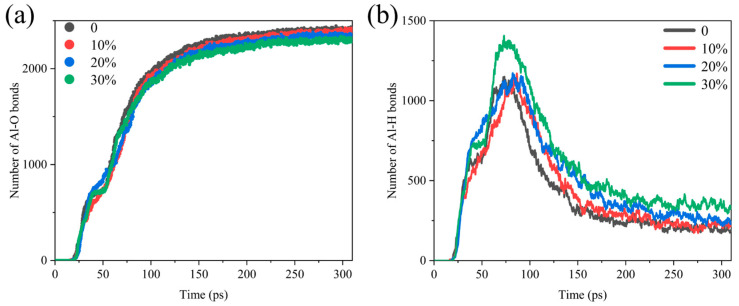
Comparative analysis of the number of (**a**) Al-O bonds and (**b**) Al-H bonds in the Al/H_2_O_2_/H_2_O system; 0, 10%, 20% and 30% represent the proportion of H_2_O in each system.

Al-O bonds and Al-H bonds are prevalent in the combustion of Al/H_2_O_2_, and the rate of its formation reflects the rate of production of aluminum oxide and aluminum hydride. Figure 2 shows the comparative analysis of the number of Al-O, and Al-H bonds in different systems, and it suggests that the generation curves of Al/H_2_O_2_/H_2_O systems with different proportions are significant differences. As shown in Figure 2a, the number of Al-O bonds reaches an extreme value later, as the water content increases. In addition, the final amount of Al-O bonds produced decreases with increasing water. The growth curve of the Al-H bond is somewhat different in that it peaks first and then declines after reaching the maximum value (Figure 2b). This indicates that the intermediate product of the reaction is the Al-H bond, which breaks after reaching a maximum value to form other products. When the time exceeds 200 ps, the number of Al-H bonds levels off, and the reaction essentially reaches equilibrium. In contrast to the evolution in the number of Al-O bonds, there is a delay in reaching the maximum value of Al-H bonds as the proportion of H_2_O to the reactants increases, and the final Al-H bonds produced increase with the water content.

H_2_O_2_ is more oxidizing than H_2_O, and the addition of water changes the reaction mechanism of the Al/H_2_O_2_ mixture. The evolution of the number of major chemical bonds in the Al/H_2_O_2_/H_2_O system with different water contents is shown in Figure S1 (Supplementary Materials). In the initial stage of the process, the decrease in the O-O bonds implies the consumption of H_2_O_2_, and the decrease followed by a slow rise in the H-O bonds implies the decomposition of H_2_O_2_ and H_2_O, with subsequent regeneration of water. We also found that, as water increased, H-H bonds became more numerous, whereas O-O bonds became less. The H-H bond is mainly associated with the formation of the product H_2_, and the source of H_2_ is formed due to the breaking of the Al-H bond. It suggests that the increase in water content increases the production of the product H_2_ by influencing the number of Al-H bonds while hindering the production of O_2_.

Analyzing the evolution of the number of products is helpful in understanding the reaction mechanism of the Al/H_2_O_2_/H_2_O system. Al and its oxides are usually found in the form of clusters. Therefore, we count them by the number of chemical bonds. Here are the equations derived from the relationship between the number of molecules and the number of bonds.

N(Al (OH)_3_) ≈ (N_bond_(O-H) − 2N(H_2_O_2_) − 2N(H_2_O) − N(•OH) − N(•OOH))/3 (1)

N(AlH_3_) = (N_bond_(Al-H))/3 (2)

In this equation, N(Al (OH)_3_), N(H_2_O_2_), N(H_2_O), N(•OH), N(•OOH), and N(AlH_3_) denote the number of molecules of Al(OH)_3_, H_2_O_2_, H_2_O, •OH, •OOH, and AlH_3_. Meanwhile Nbond(O-H) and Nbond(Al-H) represent the number of O-H and Al-H bonds, respectively. There are very few molecules (e.g., H_4_O_2_) on the right-hand side of Equation (1), which can be ignored, so Equation (1) uses “≈”. To investigate the influence of water addition on the Al/H_2_O_2_ reaction mechanism, we calculated the number of products of the Al/H_2_O_2_ system containing 30% H_2_O as a function of time through codes and equations.

**Figure 3:**
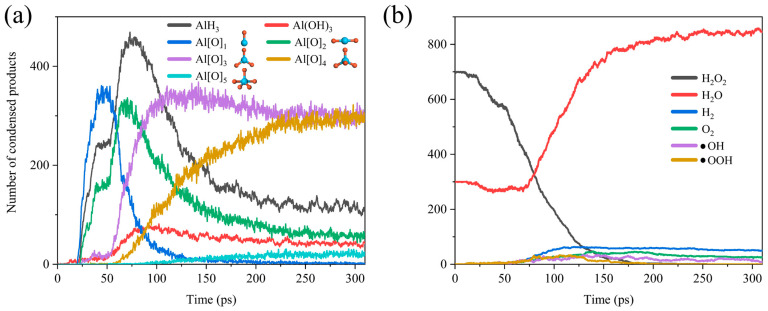
Evolution of (**a**) the products AlH_3_, Al(OH)_3_, Al[O]_x_ and the configuration of the corresponding ligand oxides of Al (Al is blue, O is red) and (**b**) gas phase chemicals H_2_O_2_, H_2_O, H_2_, O_2_, •OH, •OOH in Al/H_2_O_2_/H_2_O (30%) system.

As shown in Figure 3, we can simply divide the whole reaction process into two stages. In the early stage of the reaction, H_2_O and H_2_O_2_ were adsorbed onto the surface of the ANPs and reacted to form AlH_3_, Al (OH)_3_, and differently coordinated Al oxides (Al[O]_1_, Al[O]_2_, Al[O]_3_, Al[O]_4_, Al[O]_5_). However, over time, as the adsorption sites on ANPs gradually decreased, Al-O, Al-H, and Al-OH bonds started to compete for the adsorption sites. Since the bond energy of Al-O (501.9 ± 10.6 kJ mol^−1^) is far higher than that of Al-H (288.0 ± 13.0 kJ mol^−1^), the bonding of Al-O bonds is more stable [54]. Thus, the H atoms began to be extruded from the ANPs at about 75 ps. The gradual decrease in the amount of AlH_3_ and the steady increase in the number of Al-O bonds were accompanied by the transformation of the lower-coordinated Al oxides (Al[O]_0_ and Al[O]_1_) into higher-coordinated Al[O]_3_, Al[O]_4_, and Al[O]_5_. With sufficient simulation time, it can be predicted that, eventually, the coordination number of the majority of Al oxides will become six, consistent with the coordination number of Al in the stable oxidation product Al_2_O_3_. Figure 3b reveals that H_2_O_2_ starts to decrease from around 20 ps until it is completely consumed at around 175 ps, while H_2_O starts to decrease from around 23 ps until it reaches a minimal value at around 68 ps. This indicates that a bit of H_2_O is also involved in the combustion reaction with Al, whereas the reactivity of H_2_O is much lower than that of H_2_O_2_, and more energy has to be absorbed to reach the reaction barrier of Al/H_2_O, so the onset of the consumption of H_2_O is slightly delayed compared to that of H_2_O_2_.

**Figure 4:**
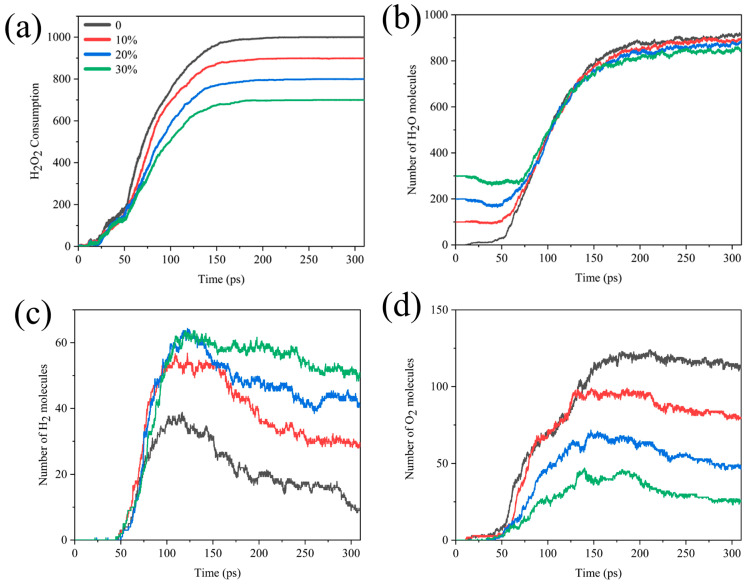
Comparison of (**a**) H_2_O_2_ molecular consumption and (**b**) H_2_O, (**c**) H_2_, (**d**) O_2_ molecular formation curves in different Al/H_2_O_2_/H_2_O systems.

Interestingly, we compared the H_2_O_2_ consumption, H_2_O, H_2_, and O_2_ formation curves for systems with different water contents. As seen in Figure 4, in the early stage of the reaction, the temperature was not too high for water to have much influence on the consumption of H_2_O_2_, but after about 50 ps, there was a difference in the rate of H_2_O_2_ consumption. The higher the proportion of H_2_O, the lower the rate of consumption of H_2_O_2_. There was a slight decrease in the amount of water early on and a rapid increase in the number of water molecules after 55 ps, until it stabilized after about 200 ps (Figure 4b). It could be assumed that Al reacted with H_2_O at the beginning of the reaction. As the reaction progressed, the system began to produce more H_2_O than the number of H_2_O molecules consumed, and the final yield of H_2_O decreased as the concentration of H_2_O_2_ decreased. Figure 4c,d correspond to the analysis of the number of bonds in Figure S1, indicating that the increase in water content increases the production of the final product H_2_ while hindering the production of O_2_.

### 3.2. Atomic Perspective of the Reaction Mechanism of ANPs/H_2_O_2_/H_2_O

**Figure 5:**
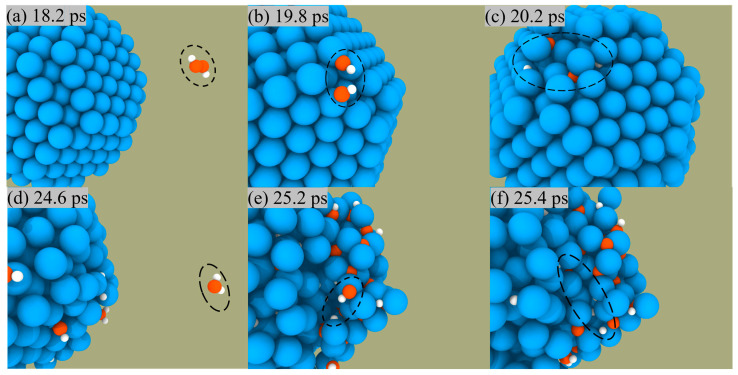
Snapshots of the reaction between H_2_O_2_ and the surface of ANPs (**a**–**c**), snapshots of the reaction between H_2_O and the surface of ANPs (**d**–**f**) in Al/H_2_O_2_/H_2_O (30%) system.

We can observe the combustion of Al/H_2_O_2_ from an atomistic perspective by capturing the microscopic trajectory of the reaction. Figure 5 presents a snapshot of the reaction of H_2_O_2_ and H_2_O on the ANPs surface in the Al/H_2_O_2_ system with 30% water. We found that both H_2_O_2_ and H_2_O molecules were constantly approaching the ANPs and then were adsorbed on the surface. During this process, H_2_O_2_ and H_2_O molecules were chemically activated, whereby the molecules decomposed into OH radicals and H radicals and were captured by ANPs. The Al-OH bond was unstable and quickly broke to form an Al-H bond with an Al-O bond. Hence, the number of Al-H and Al-O bonds was much higher than the number of Al-OH bonds as the simulation time increased.

**Figure 6:**
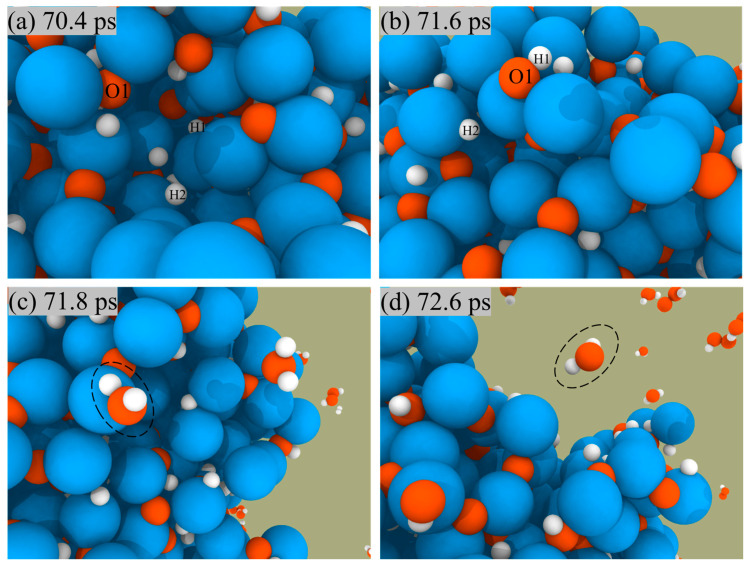
Snapshot of H_2_O formation (**a**–**c**) and desorption (**d**) from the surface of ANPs in Al/H_2_O_2_/H_2_O (30%) system.

ANPs and H_2_O_2_ are used as bipropellants mainly owing to the fact that the products of the reaction are green and non-polluting, so we focus on analyzing the generation routes of their products: H_2_O, H_2,_ and O_2_. Figure 6 shows a snapshot of H_2_O generation from the surface of ANPs. We could see that the reaction starts with the O and H atoms on the ANPs, approaching each other to form Al-OH bonds, then combining with the H on the nearby ANPs to form H_2_O, which is eventually desorbed from the surface of the ANPs.

**Figure 7:**
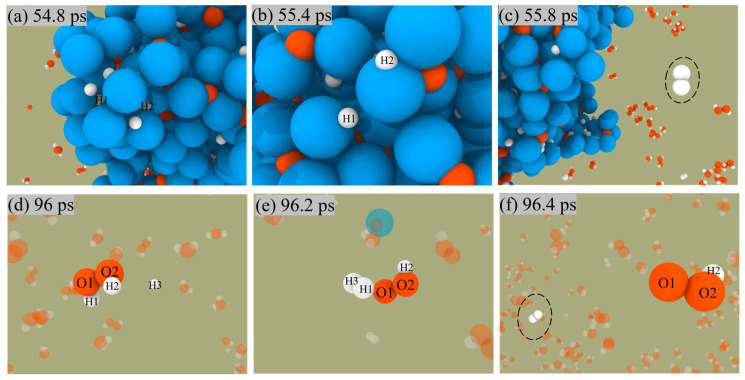
Snapshot of H_2_ generation from ANPs surface (**a**–**c**) and solution (**d**–**f**) in Al/H_2_O_2_/H_2_O (30%) system.

The two paths for producing H_2_ are shown in Figure 7. In the first pathway, the H atoms on the surface of ANPs come close to each other, then form H-H bonds, and finally release from the surface of ANPs to generate H_2_ molecules. The other mechanism is that the free H radical approaches the H_2_O_2_ molecule in solution and then combines with an H of the H_2_O_2_ molecule to form an H_2_ molecule and a peroxide hydroxyl radical (•OOH). The reaction proceeds as in Equation (3).

H + H_2_O_2_ → H_2_ + HO_2_ (3)

Since the number of H radicals is relatively small, H_2_ is mainly generated by the first pathway.

**Figure 8:**
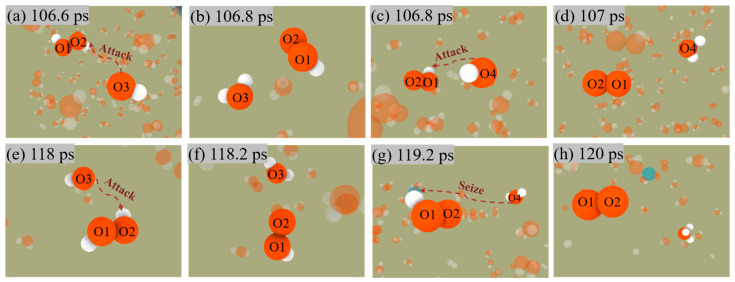
Snapshot of O_2_ generation by the first mechanism (**a**–**d**) and the second mechanism (**e**–**h**) in Al/H_2_O_2_/H_2_O (30%) system.

Similarly, Figure 8 shows the two pathways for generating O_2_. Nevertheless, unlike the production of H_2_, O_2_ does not release from the surface of ANPs, which also indicates that the Al-O bond is much more stable than the Al-H bond.

H_2_O_2_ + OH → H_2_O + HO_2_ (4)

HO_2_ + OH → H_2_O + O_2_ (5)

HO_2_ + H_2_O → H_3_O + O_2_ (6)

Both mechanisms have the same reaction in the first stage, i.e., Equation (4), in which OH radicals in solution attack the H_2_O_2_ molecule to form an H_2_O molecule and an OOH radical. The difference is that in the first mechanism, i.e., Equation (5), the OH radical attacks the OOH radical again followed by the generation of an H_2_O molecule and an O_2_ molecule. The second mechanism, Equation (6), involves the H_2_O molecule continually approaching the OOH radical and then seizing one of its H, eventually forming an H_3_O radical with an O_2_ molecule.

### 3.3. Adiabatic Combustion Processes in the ANPs/H_2_O_2_/H_2_O System

**Figure 9:**
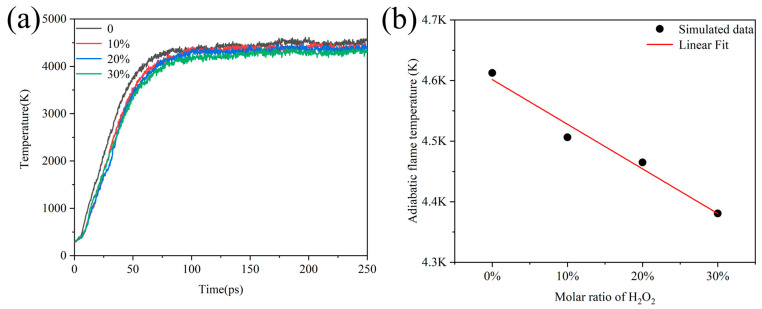
(**a**) Evolution of adiabatic flame temperature over time for Al/H_2_O_2_/H_2_O systems with different percentages and (**b**) linear fitting of adiabatic flame temperature to the ratio of H_2_O under steady state combustion.

Under experimental conditions, H_2_O_2_ usually contains water in varying proportions, so we wanted to investigate the influence of the addition of H_2_O on the temperature at which Al/H_2_O_2_ burned. The simulation under the NVT ensemble and constant heating rate cannot reflect the influence of H_2_O on combustion. To address this problem, we simulated 250 ps with an NVE ensemble to obtain the adiabatic flame temperature of the combustion. Figure 9a shows the evolution of temperature over time for systems containing different proportions of H_2_O. We considered the equilibrium temperature at the end of the simulation as the adiabatic flame temperature of the combustion system. Then, it was found that the temperature of the systems all reached extreme values after 100 ps and remained stable, while the temperature of the adiabatic flame decreased with the increase in the percentage of H_2_O. This means that the adiabatic flame temperature decreased from 4612 K to 4380 K as the percentage of water increased from 0 to 30%.

### 3.4. Influence of the Heating Speed on the Combustion of the System

**Figure 10:**
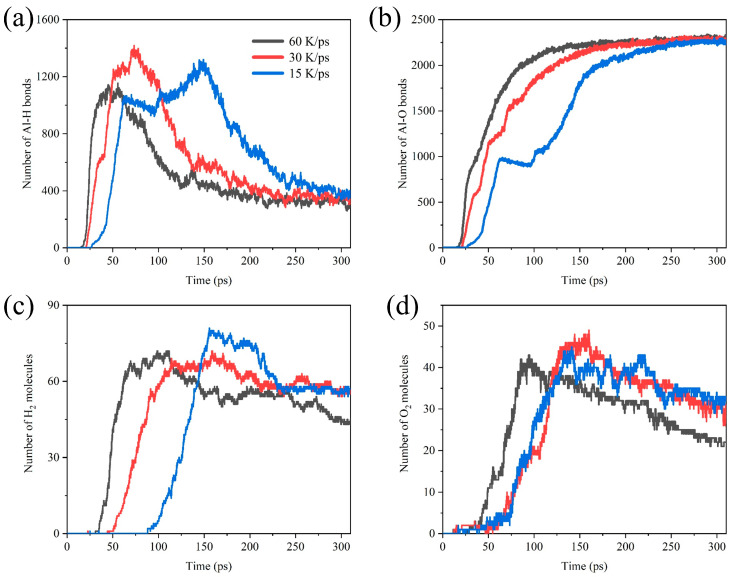
Comparative analysis of the number of (**a**) Al-H bonds, (**b**) Al-O bonds, (**c**) H_2_ molecules, and (**d**) O_2_ molecules in the Al/H_2_O_2_/H_2_O (30%) system with different heating speeds.

To obtain reliable results of the simulations, we simulated the Al/H_2_O_2_/H_2_O (30%) system with 60 K/ps, 30 K/ps, and 15 K/ps heating speed. Figure 10 shows the comparative analysis of the number of Al-H bonds, Al-O bonds, H_2_, and O_2_ in systems with different heating speeds. As shown in Figure 10a,b, in the early stages of the reaction, the rate of Al-O and Al-H bond formation is positively correlated with the heating speed. The peak of the Al-H bonds is also delayed as the heating speed decreases. We also found a plateau in Al-O bonds formation at a heating rate of 15 K/ps. It is not difficult to notice that the rate of early Al-O bonds formation actually decreases briefly at 30 K/ps and 60 K/ps heating rates. This may be due to the fact that the final number of Al-O bonds is close to each other at temperatures of 750 K~1500 K, and the lower heating rate amplifies the appearance of this plateau period. At the heating rate of 60 K/ps, the yield of H_2_ molecules is significantly reduced. This is attributed to the fact that the system reaches high temperatures early, prompting the reaction between H_2_ molecules and O_2_ molecules to form H_2_O.

### 3.5. Influence of the Oxide Layer on the Combustion of the System

Considering the passivation of Al in the natural environment, we coated the surface of ANPs with an oxide layer in the simulation. Al particles with a diameter of 2.8 nm were placed in a periodic box of size 10.0 nm × 10.0 nm × 10.0 nm containing 200 O_2_ molecules and then subjected to NVT simulations at 200 K for 50 ps, cycling six times. The detailed process is shown in Figure S2. Eventually, the Al_675_O_314_/H_2_O_2_/H_2_O system was obtained. The Al_675_O_314_/H_2_O_2_/H_2_O system was then simulated under the same simulation conditions as the Al/H_2_O_2_/H_2_O system.

**Figure 11:**
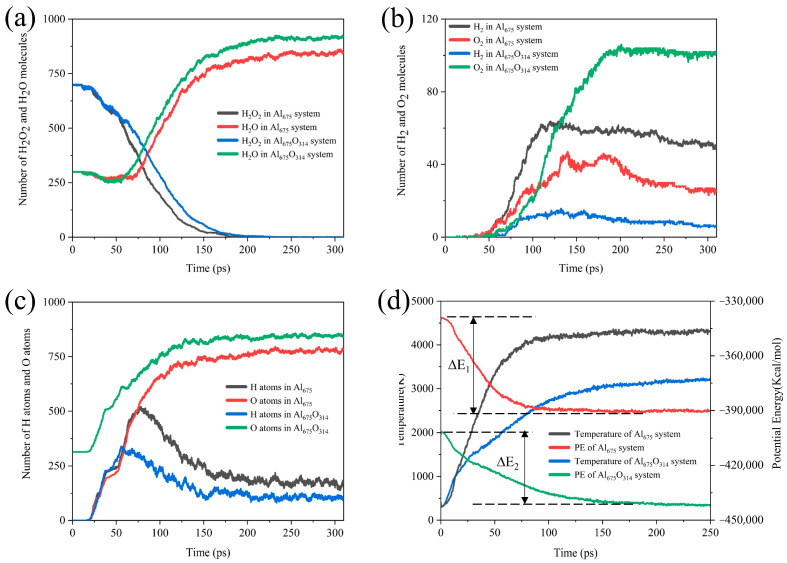
Evolution of (**a**) the amount of H_2_O_2_/H_2_O, (**b**) H_2_/O_2_, (**c**) the H and O atoms on ANPs over time, and (**d**) the simulated temperature and potential energy over time under adiabatic conditions in the Al_675_/H_2_O_2_/H_2_O (30%) system and Al_675_O_314_/H_2_O_2_/H_2_O (30%) system.

As shown in Figure 11, we found that the coverage of the oxide layer resulted in a decrease in the reactive aluminum content of the ANPs, so the rate of H_2_O_2_ consumption decreased. Due to the existence of the oxide layer and the fact that the Al-O bond is more stable than the Al-H bond, the O atoms occupied the reaction site of the ANPs earlier, resulting in the H atoms on the ANPs reaching a maximum value earlier, and the H content on the Al was lower. Since the release of H_2_ from the surface of ANPs is the most dominant source of H_2_ in the product, ANPs that have been passivated contain more O atoms. Hence, the reaction favored the evolution of H_2_O from the surface of ANPs over the production of H_2_. The yield of H_2_O increased in the systems with an oxide layer, while the yield of H_2_ became poor. Understandably, due to the passivation of Al in the initial stage, the active aluminum content of the ANPs was reduced, the Al_675_O_314_/H_2_O_2_/H_2_O system was less energetic, the reaction released less energy (22% less compared to Al_675_/H_2_O_2_/H_2_O (30%) system), and the adiabatic flame temperature decreased from 4357 K to 3256 K.

**Figure 12:**
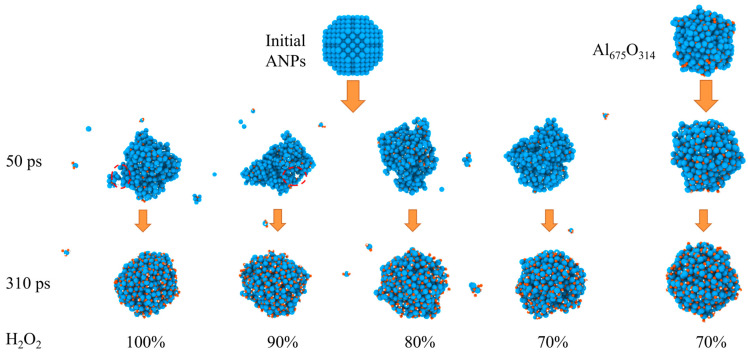
Evolution of the morphology of ANPs in systems with different molar ratios of Al/H_2_O_2_/H_2_O and in systems with an oxide layer (Al, oxygen, and hydrogen are blue, orange, and white, respectively).

**Figure 13:**
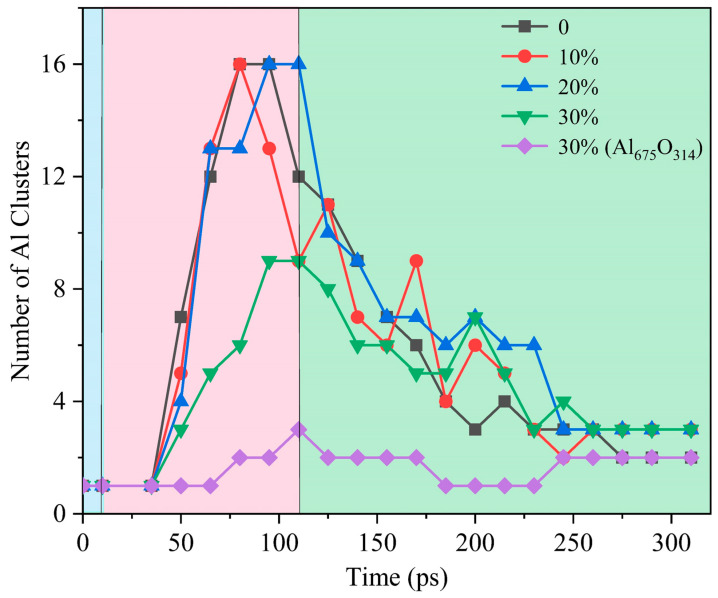
Evolution of the number of Al clusters in five systems (blue background for the configuration optimization phase, pink background for the heating phase, green background for the thermostatic phase).

The evolution of the morphology of ANPs with different molar ratios of Al/H_2_O_2_/H_2_O systems and systems with oxide layer is shown in Figure 12. In connection with Figure 13, we found that the violent combustion of ANPs in the H_2_O_2_/H_2_O system also changed its morphology. The temperature of the system gradually increased with time, and the epitaxial growth of the chain-like structure of ANPs became more apparent, accompanied by an increase in the internal voids. When the temperature reached the melting point of Al (about 933 K), many small fragments of Al clusters were also generated. However, as the system temperature continued to increase, the chain-like structure of ANPs gradually disappeared, the volume contracted, agglomeration occurred, and the number of Al clusters gradually decreased. The addition of H_2_O also influences the morphological evolution of ANPs. At 50 ps, the morphology of ANPs in the Al/H_2_O_2_/H_2_O system with 30% H_2_O was only slightly deformed. As the H_2_O content decreased, the deformation of ANPs increased and even cavities were formed. In addition, the ANPs would also separate to form more and more small fragments. While the addition of the oxide layer reduced the reaction energy release of the system, the ANPs deformed even less and basically did not rupture or decompose into small fragments.

## 4. Conclusions

In summary, we simulated the effect of H_2_O addition on Al/H_2_O_2_ combustion by using the ReaxFF force field. Here are several significant findings and conclusions from this paper:

(1) With the percentage of H_2_O increased from 0 to 30%, the number of Al-O bonds on the ANPs decreases, and the number of Al-H bonds increases. Meanwhile, the increase in the water content would increase the production of the final product H_2_ and hinder the production of O_2_.

(2) The combustion mechanism of the Al/H_2_O_2_/H_2_O system was investigated from an atomistic perspective. H_2_O_2_ molecules and H_2_O molecules were adsorbed on the surface of ANPs, then chemically activated and decomposed into OH radicals and H radicals, which were finally captured by ANPs. The Al-O bond is more stable than the Al-H bond, which is why H_2_O and H_2_ could be generated and desorbed from the ANPs surface, while O_2_ could not be generated through this pathway. H and O would compete for the reaction sites of ANPs, whereby the number of Al-O bonds would be far higher than that of Al-H and Al-OH bonds as the simulation proceeds, and Al[O]_x_ would be converted from low to high coordination.

(3) The effect of the content of H_2_O on the adiabatic flame of the Al/H_2_O_2_/H_2_O system was investigated. The results showed that the adiabatic flame temperature decreased from 4612 K to 4380 K as the percentage of water increased from 0 to 30%.

(4) In addition, the influence of different heating speed on the combustion of the system was investigated. The simulation results indicated that the heating speed only affected the early formation of Al-O and Al-H bonds, but not their final number. A high heating rate promoted the reaction between the H_2_ molecules and the O_2_ molecules to form H_2_O.

(5) Finally, the combustion of passivated ANPs with H_2_O_2_/H_2_O was simulated. The coating of the oxide layer would reduce the rate of H_2_O_2_ consumption and H_2_ production significantly while preventing drastic deformation of the ANPs’ morphology.

## Figures and Tables

**Table 1:** Model parameters of the simulation cells.

Molar Ratio (H_2_O)	System Component	Size (Angstrom^3^)	Total Atoms
0	1000H_2_O_2_ + 675Al	100 × 100 × 100	4675
10%	900H_2_O_2_ + 100H_2_O + 675Al	100 × 100 × 100	4575
20%	800H_2_O_2_ + 200H_2_O + 675Al	100 × 100 × 100	4475
30%	700H_2_O_2_ + 300H_2_O + 675Al	100 × 100 × 100	4375

## Data Availability

Data are contained within the article or Supplementary Materials.

## Supplementary Materials

The following supporting information can be downloaded at: https://www.mdpi.com/article/10.3390/molecules29071567/s1. Figure S1: Evolution of the number of H-H, H-O, Al-H, Al-O, O-O bonds for each system. Figure S2: Formation of oxide layer on the surface of ANPs.

## References

[1] Zhang Q.H., Shreeve J.M. (2013). Ionic liquid propellants: Future fuels for space propulsion. Chem. Eur. J..

[2] Gohardani A.S., Stanojev J., Demaire A., Anflo K., Persson M., Wingborg N., Nilsson C. (2014). Green space propulsion: Opportunities and prospects. Prog. Aerosp. Sci..

[3] Nosseir A.E.S., Cervone A., Pasini A. (2021). Review of state-of-the-art green monopropellants: For propulsion systems analysts and designers. Aerospace.

[4] Barato F. (2023). Review of alternative sustainable fuels for hybrid rocket propulsion. Aerospace.

[5] Santos L.B., Ribeiro C.A., Capela J.M.V., Crespi M.S., Pimentel M.A.S., De Julio M. (2013). Kinetic parameters for thermal decomposition of hydrazine. J. Therm. Anal. Calorim..

[6] Kumar P. (2019). Advances in phase stabilization techniques of AN using KDN and other chemical compounds for preparing green oxidizers. Def. Technol..

[7] Guseinov S.L., Fedorov S.G., Kosykh V.A., Storozhenko P.A. (2020). Hydrogen peroxide decomposition catalysts used in rocket engines. Russ. J. Appl. Chem..

[8] Jung S., Choi S., Heo S., Kwon S. (2021). Scaling of catalyst bed for hydrogen peroxide monopropellant thrusters using catalytic decomposition modeling. Acta Astronaut..

[9] Okninski A., Surmacz P., Bartkowiak B., Mayer T., Sobczak K., Pakosz M., Kaniewski D., Matyszewski J., Rarata G., Wolanski P. (2021). Development of green storable hybrid rocket propulsion technology using 98% hydrogen peroxide as oxidizer. Aerospace.

[10] Markandan K., Chin J.K., Cheah K.H., Tan M.T.T. (2018). Recent developments in ceramic microthrusters and the potential applications with green propellants: A review. Clean Technol. Environ. Policy.

[11] Wang Z.D., Herbinet O., Hansen N., Battin-Leclerc F. (2019). Exploring hydroperoxides in combustion: History, recent advances and perspectives. Prog. Energy Combust. Sci..

[12] Parzybut A., Surmacz P., Gut Z. (2023). Impact of hydrogen peroxide concentration on manganese oxide and platinum catalyst bed performance. Aerospace.

[13] Cong Y., Zhang T., Li T., Suo J.W., Wang X.D., Ma L., Liang D.B., Lin L.W. (2004). Propulsive performance of a hypergolic H_2_O_2_/kerosene bipropellant. J. Propuls. Power.

[14] Li X.T., Tian H., Yu N.J., Cai G.B. (2014). Experimental investigation of fuel regression rate in a HTPB based lab-scale hybrid rocket motor. Acta Astronaut..

[15] Tian H., Sun X.L., Guo Y.D., Wang P.F. (2015). Combustion characteristics of hybrid rocket motor with segmented grain. Aerosp. Sci. Technol..

[16] Li S., Ge Y.F., Wei X.L., Li T. (2016). Mixing and combustion modeling of hydrogen peroxide/kerosene shear-coaxial jet flame in lab-scale rocket engine. Aerosp. Sci. Technol..

[17] Li H.X., Ye L., Wei X.L., Li T., Li S. (2017). The design and main performance of a hydrogen peroxide/kerosene coaxial-swirl injector in a lab-scale rocket engine. Aerosp. Sci. Technol..

[18] John J., Nandagopalan P., Baek S.W., Cho S.J. (2020). Hypergolic ignition delay studies of solidified ethanol fuel with hydrogen peroxide for hybrid rockets. Combust. Flame.

[19] Okninski A. (2018). On use of hybrid rocket propulsion for suborbital vehicles. Acta Astronaut..

[20] Starik A.M., Savel’ev A.M., Titova N.S. (2015). Specific features of ignition and combustion of composite fuels containing aluminum nanoparticles (Review). Combust. Explos. Shock. Waves.

[21] Sundaram D.S., Yang V., Zarko V.E. (2015). Combustion of nano aluminum particles (Review). Combust. Explos. Shock. Waves.

[22] DeLuca L.T. (2018). Overview of Al-based nanoenergetic ingredients for solid rocket propulsion. Def. Technol..

[23] Vadhe P.P., Pawar R.B., Sinha R.K., Asthana S.N., Rao A.S. (2008). Cast aluminized explosives. Combust. Explos. Shock. Waves.

[24] Kim Y., Park Y., Yoh J.J. (2019). Slow and rapid thermal decomposition characteristics of enhanced blast explosives for burning in fuel-rich, oxygen-rich conditions. Thermochim. Acta.

[25] Pang W.Q., Fan X.Z., Wang K., Chao Y.M., Xu H.X., Qin Z., Zhao F.Q. (2020). Al-based nano-sized composite energetic materials (Nano-CEMs): Preparation, characterization, and performance. Nanomaterials.

[26] Jayaraman K., Sivakumar P.M., Zarrabi A., Sivakumar R., Jeyakumar S. (2021). Combustion characteristics of nanoaluminium-based composite solid propellants: An overview. J. Chem..

[27] He Q.Q., Wang J., Mao Y.F., Cao W., Chen J., Nie F.D. (2022). An effective strategy to improve combustion and pressure output performance of HMX/Al. Combust. Flame.

[28] Sabourin J.L., Risha G.A., Yetter R.A., Son S.F., Tappan B.C. (2008). Combustion characteristics of nanoaluminum, liquid water, and hydrogen peroxide mixtures. Combust. Flame.

[29] Zaseck C.R., Son S.F., Pourpoint T.L. (2013). Combustion of micron-aluminum and hydrogen peroxide propellants. Combust. Flame.

[30] Schmitt M.M., Bowden P.R., Tappan B.C., Henneke D. (2018). Steady-state shock-driven reactions in mixtures of nano-sized aluminum and dilute hydrogen peroxide. J. Energetic Mater..

[31] Sundaram D.S., Yang V. (2014). Combustion of micron-sized aluminum particle, liquid water, and hydrogen peroxide mixtures. Combust. Flame.

[32] Chu Q.Z., Shi B.L., Liao L.J., Luo K.H., Wang N.F., Huang C.G. (2018). Ignition and oxidation of core-shell Al/Al_2_O_3_ nanoparticles in an oxygen atmosphere: Insights from molecular dynamics simulation. J. Phys. Chem. C.

[33] Ashraf C., van Duin A.C.T. (2017). Extension of the ReaxFF combustion force field toward syngas combustion and initial oxidation kinetics. J. Phys. Chem. A.

[34] Zeng H.D., Cheng X.L., Zhang C.Y., Lu Z.P. (2018). Responses of core-shell Al/Al**_2_**O_3_ nanoparticles to heating: ReaxFF molecular dynamics simulations. J. Phys. Chem. C.

[35] Hong D.K., Li Z.H., Si T., Guo X. (2020). A study of the effect of H_2_O on char oxidation during O_2_/H_2_O combustion using reactive dynamic simulation. Fuel.

[36] Liu J.P., Liu P.G., Wang M.J., Wang W.C., Lv F.W., Sun R.C., Yang Y.X. (2020). Combustion of Al nanoparticles coated with ethanol/ether molecules by non-equilibrium molecular dynamics simulations. Mater. Today Commun..

[37] Cheng Y.X., Zhao Y., Zhao F.Q., Xu S.Y., Ju X.H., Ye C.C. (2022). ReaxFF simulations on the combustion of Al and n-butanol nanofluid. Fuel.

[38] Bai Z.Z., Jiang X.Z., Luo K.H. (2023). Understanding mechanisms of pyridine oxidation with ozone addition via reactive force field molecular dynamics simulations. Chem. Eng. Sci..

[39] Li G., Niu L.L., Hao W.Z., Liu Y., Zhang C.Y. (2020). Atomistic insight into the microexplosion-accelerated oxidation process of molten aluminum nanoparticles. Combust. Flame.

[40] Zhao Y., Ma D.X., Zhao F.Q., Xu S.Y., Ju X.H. (2022). Atomic insights into the combustion behavior of Al nano-droplets with H_2_O vapor at high temperature. Appl. Surf. Sci..

[41] Hao W.Z., Li G., Niu L.L., Gou R.J., Zhang C.Y. (2020). Molecular dynamics insight into the evolution of Al nanoparticles in the thermal decomposition of energetic materials. J. Phys. Chem. C.

[42] Zhao Y., Mei Z., Zhao F.Q., Xu S.Y., Ju X.H. (2021). Atomic perspectives revealing the evolution behavior of aluminum nanoparticles in energetic materials. Appl. Surf. Sci..

[43] van Duin A.C.T., Dasgupta S., Lorant F., Goddard W.A. (2001). ReaxFF: A reactive force field for hydrocarbons. J. Phys. Chem. A.

[44] Chenoweth K., van Duin A.C.T., Goddard W.A. (2008). ReaxFF reactive force field for molecular dynamics simulations of hydrocarbon oxidation. J. Phys. Chem. A.

[45] Senftle T.P., Hong S., Islam M.M., Kylasa S.B., Zheng Y.X., Shin Y.K., Junkermeier C., Engel-Herbert R., Janik M.J., Aktulga H.M. (2016). The ReaxFF reactive force-field: Development, applications and future directions. npj Comput. Mater..

[46] Plimpton S. (1995). Fast parallel algorithms for short-range molecular-dynamics. J. Comput. Phys..

[47] Aktulga H.M., Fogarty J.C., Pandit S.A., Grama A.Y. (2012). Parallel reactive molecular dynamics: Numerical methods and algorithmic techniques. Parallel Comput..

[48] Hong S., van Duin A.C.T. (2016). Atomistic-scale analysis of carbon coating and its effect on the oxidation of aluminum nanoparticles by ReaxFF-molecular dynamics simulations. J. Phys. Chem. C.

[49] Li G., Niu L.L., Xue X.G., Hao W.Z., Liu Y., Zhang C.Y. (2020). Atomic perspective about the reaction mechanism and H_2_ production during the combustion of Al nanoparticles/H_2_O_2_ bipropellants. J. Phys. Chem. A.

[50] Dong R.K., Mei Z., Zhao F.Q., Xu S.Y., Ju X.H. (2021). Initial oxidation of nano-aluminum particles by H_2_O/H_2_O_2_: Molecular dynamics simulation. Int. J. Hydrogen Energy.

[51] Martínez L., Andrade R., Birgin E.G., Martínez J.M. (2009). PACKMOL: A package for building initial configurations for molecular dynamics simulations. J. Comput. Chem..

[52] Zheng M., Li X.X., Wang M.J., Guo L. (2019). Dynamic profiles of tar products during Naomaohu coal pyrolysis revealed by large-scale reactive molecular dynamic simulation. Fuel.

[53] Stukowski A. (2010). Visualization and analysis of atomistic simulation data with OVITO-the Open Visualization Tool. Model. Simul. Mater. Sci. Eng..

[54] Luo Y.R., Kerr J.A. (2012). Bond Dissociation Energies.

