# Supplementary material for: Atomistic Insights into the Influence of High Concentration H2O2/H2O on Al Nanoparticles Combustion: ReaxFF Molecules Dynamics Simulation
Source: Molecules. 2024 Mar 31;29(7):1567. doi: 10.3390/molecules29071567 (PMC11013683; doi:10.3390/molecules29071567)
Supplement: Supplementary file 1 [file molecules-29-01567-s001.zip › molecules-2930336-supplementary.pdf]

Supporting Information

Atomistic Insights into the Influence of High Concentration H_2O_2/H_2O on Al Nanoparticles Combustion: ReaxFF molecules dynamics simulation

Xindong Yu [1], Pengtu Zhang [2], Heng Zhang [1]* and Shiling Yuan[1]*

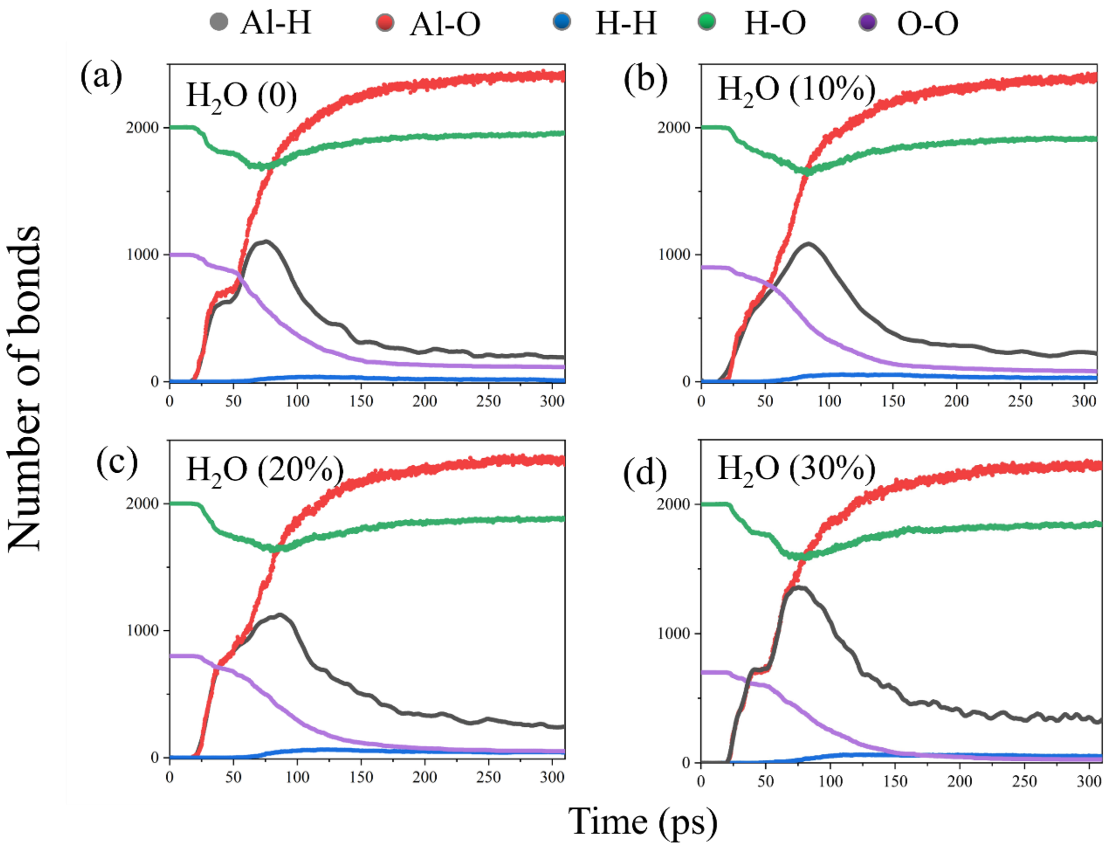

Figure S1. Evolution of the number of H-H, H-O, Al-H, Al-O, O-O bonds for each system.

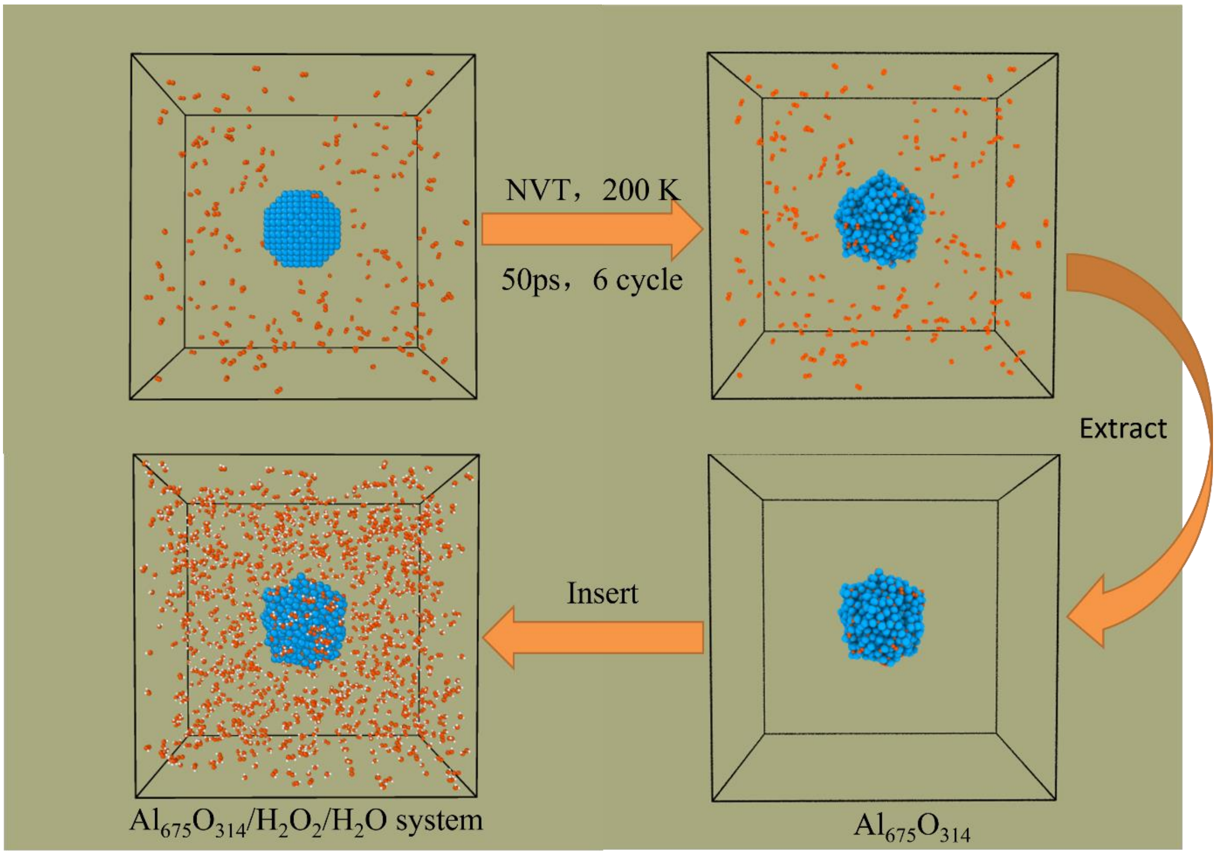

Figure S2. Formation of oxide layer on the surface of ANPs.
